# Impact of regenerative procedure on the healing process following surgical root canal treatment: A systematic review and meta-analysis

**DOI:** 10.1371/journal.pone.0312751

**Published:** 2025-01-02

**Authors:** Nader Muthanna, Xiaoyue Guan, Fouad Alzahrani, Badr Sultan Saif, Abdelrahman Seyam, Ahmed Alsalman, Ahmed Es Alajami, Ang Li

**Affiliations:** 1 Key Laboratory of Shaanxi Province for Craniofacial Precision Medicine Research, College of Stomatology, Xi’an Jiaotong University, Xi’an, Chin; 2 Department of Endodontic, College of Stomatology Xi’an Jiaotong University, Xi’an, China; 3 Pulp Biology and Endodontic Department, Al-Baha Dental Center, Al-Baha, Saudi Arabia; 4 Department of Orthodontics, College of Stomatology, First Affiliated Hospital of Xi’an Jiaotong University, Xi’an, China; 5 Department of Oral Preventive, College of Stomatology Xi’an Jiaotong University, Xi’an, China; 6 Department of Periodontology, College of Stomatology, Xi’an Jiaotong University, Xi’an, China; Aga Khan University Hospital, PAKISTAN

## Abstract

**Introduction:**

Different Guided Tissue Regeneration (GTR) procedures, such as membranes, bone substitute materials, and Autologous Platelet Concentrates (APCs), have been applied after surgical root canal treatment (SRCT), which produce different outcomes. This study aimed to evaluate the impact of regenerative procedures on the healing process following SRCT.

**Methods:**

A comprehensive search of PubMed, Embase, Scopus, Cochrane, and the Web of Science found Randomized Controlled Trials (RCTs) published until February 25, 2024. Manual searches were also conducted. Our main outcome was SRCT success or failure after GTR procedures. The Risk Ratio (RR) and failure rate meta-analysis used a fixed effects model with a 95% confidence interval (CI). Subgroup analyses were conducted based on the use of different GTR procedures for varying lesion types in SRCT.

**Results:**

Out of 1,605 records, 16 studies with 690 lesions were included. Overall, GTR procedures significantly improved healing after SRCT in both 2D (RR: 0.50; 95% CI, 0.34–0.73; P < 0.001) and 3D evaluation methods (RR: 0.36; 95% CI, 0.15–0.90; P < 0.001) with no significant difference between the two methods.

**Conclusion:**

GTR significantly improved SRCT healing regardless of the evaluation method used. Combining collagen membranes with bovine bone-derived hydroxyapatite significantly enhanced the healing process. Additionally, GTR procedures significantly improve healing in through-and-through lesions.

## Introduction

Bacterial infection can result in pulpal inflammation, ultimately leading to pulp necrosis and periapical lesions [1]. While conventional Root Canal Treatment (RCT) is the first-line treatment, Surgical Root Canal Treatment (SRCT) is recommended in cases where non-surgical RCT is unsuccessful [2, 3]. The main objective of SRCT is to create an optimal environment for periapical tissue repair. This is typically achieved by eliminating infections and inaccessible areas within the root canal system and preventing future infections [4].

Success in both RCT and SRCT relies on the absence of signs of infection and inflammation, along with radiography showing reduced periapical lesion size and normal growth of the periodontal ligament gap [5]. The evaluation of healing after SRCT is commonly conducted using the criteria established by Rud et al.[6] and Molven et al. on 2D imaging [7]. On the other hand, the Modified PENN 3D criteria have been used to evaluate healing on 3D imaging[8].

Wound healing after SRCT can result in repair or regeneration. Repair involves the development of new tissue that differs from the original cells, while regeneration involves wound healing using cells from a similar tissue [9]. However, the potential for connective tissue to invade the bony defect can interfere with the healing process [10]. Guided Tissue Regeneration (GTR) procedures have shown promise in periodontology and dental implants and have gained interest as a supplementary approach for SRCT to improve healing and prevent soft tissue collapse within the bony defect [11, 12].

Different materials for GTR can be used in SRCT, including barrier membranes, bone grafts, and Autologous Platelet Concentrates (APCs), either alone or in combination [4]. The first commercially produced material was expanded polytetrafluoroethylene (e-PTFE), but its complete removal requires additional surgery [13]. Resorbable membranes, like collagen membranes, were developed in the 1990s to avoid the need for surgical removal [14].

Different types of bone grafts offer unique advantages, disadvantages, and success rates. Familiar sources include autogenous, xenograft, allograft, and alloplast [12]. The gold standard for bone grafting is autogenous grafts for their ability to promote osteogenesis, osteoinduction, and osteoconduction characteristics [15]. However, they have disadvantages such as longer surgical time, morbidity, and limited bone supply [16]. Xenograft bone, taken from animals like bovines, is becoming more popular for its osteoconductive properties [17, 18]. Allograft bone, donated between genetically dissimilar individuals, can be osteoconductive or osteoinductive without additional surgery. Alloplasts are synthetic materials considered exclusively osteoconductive [19].

GTR using APCs showed high amounts of cytokines and growth factors, making it a promising option for tissue regeneration [20]. These cells are obtained from the patient’s peripheral blood, making the procedure safer, well-tolerated, and cheaper [20]. Meanwhile, platelet-rich plasma (PRP), the first generation of APCs, is difficult to prepare and requires anticoagulants [21–23]. The second generation of platelet-rich fibrin (PRF) can be obtained through a single centrifugation method [24]. A third-generation injectable PRF (i-PRF) was developed in 2014 using a different centrifugation force and plastic tubes, reducing clotting time [25].

Many studies have evaluated the efficacy of GTR procedures[26–28]. However, there is still debate about their impact on improving success rates after SRCT. Therefore, a systematic review and meta-analysis of these studies are necessary to aid clinicians in making informed decisions for successful SRCT. This study aimed to evaluate the impact of GTR procedures on the healing process following SRCT.

## Material and methods

The systematic review and meta-analysis followed PRISMA 2020 [29] and the Cochrane Handbook for Systematic Reviews of Interventions [30]. The systematic review process was registered in PROSPERO (CRD 42023477089). The PICOST strategy was utilized to formulate the clinically relevant question: Among individuals having Surgical Root Canal Treatment (P), will the application of a GTR procedure (I) versus not using a GTR procedure (C) have an impact on the healing process (O) in randomized clinical trials (S) after one year follow up (T)?

### Search strategy

The online search was carried out independently by three researchers (N.M., X.G., and A.S.) to locate relevant studies. The electronic databases PubMed, Embase, Scopus, Cochrane, and Web of Science were searched until February 25, 2024. Grey literature was found using Google Scholar and OpenGrey. No restrictions were placed on the publication date or language. Specific keywords were merged using Boolean operators, and the MeSH terms were incorporated into the electronic search strategy (S1 Table). In addition, manual searches were also conducted by checking the reference lists of relevant articles.

### Inclusion and exclusion criteria

This systematic review compared randomized clinical trials that used GTR procedures in the intervention group to a control group that did not. The trials had to have a minimum follow-up of 12 months and focus on periapical lesions caused by endodontic problems in human patients. Clinical assessment was based on signs and symptoms, while radiographic assessment used criteria established by Molven et al. [7] or Rud et al. [6] for 2D imaging, and modified PENN 3D criteria by Schloss et al. [8] for 3D imaging. Eligible patients had to be classified as American Society of Anesthesiologists (ASA) I or II. Studies were excluded if they did not have sufficient data, included patients with root fractures, resorption, or perforation, or included children under 12 or with sample sizes less than 10.

### Study selection

The records were imported into EndNote X21 (Clarivate Analytics, Philadelphia, PA). After the removal of duplicates, the titles and abstracts of the remaining records were screened independently for eligibility by 3 reviewers (N.M., X.G., and B.S.). The same three authors read the full texts of articles independently to determine if they met the inclusion criteria. References of all relevant research were also checked. A fourth reviewer (A.L.) was consulted to facilitate compromise in any disagreement.

### Data extraction

Three independent reviewers (N.M., X.G., and F.A.) Obtained data from every study using a standardized Excel spreadsheet. The form included the following information for each study: the first author’s name, the year of publication, the age, the size or type of lesion, the regenerative techniques and materials used, the sample size, and the outcomes observed at the 12-month follow-up. Studies with missing outcome data were excluded.

The clinical outcomes were evaluated by the presence or absence of signs of infection and inflammation. Radiographically, the healing assessment was determined by using the criteria established by Rud et al. [6] or Molven et al. [7] (complete, incomplete, uncertain, or unsatisfactory healing) for 2D imaging evaluation, whereas the modified PENN 3D criteria established by Schloss et al. [8] (complete, limited, uncertain, or unsatisfactory healing) were used for 3D imaging evaluation.

The assessment of success and failure was determined based on a comprehensive evaluation of both clinical and radiological outcomes. For statistical purposes, the outcomes were also dichotomized into success and failure. The success was referred to the loss of clinical symptoms and the signs of (complete or incomplete healing) for 2D imaging and (Complete or Limited healing) for 3D imaging. Failure was referred to the presence of clinical symptoms and/or the occurrence of (uncertain or unsatisfactory healing) for 2D and 3D imaging.

### Quality assessment

N.M., B.S., and F.A. evaluated the risk of bias in each study using the Cochrane Collaboration tool for randomized trials (RoB 2). They assessed trials using RoB 2 questions and determined each study’s risk of bias using the algorithm in the RoB 2 guidance. Each study’s overall risk of bias was assessed by considering each domain’s risk. If all domains of the study were low risk, the study was evaluated as having a low risk of bias overall. If the study had some concerns in at least one domain but no high risk, the study was evaluated as having some concerns of bias overall. If the study had a high risk in one or more domains, it was considered to have a high risk of bias overall. Disagreements amongst reviewers were resolved through discussion. Otherwise, a fourth reviewer, A.L., was consulted until agreement was reached.

### Statistical analysis

The Cochrane Collaboration System’s Review Manager 5.4 was utilized to calculate the risk ratio (RR) to compare SRCT failures with and without GTR treatments. The chi-squared test (X2) assessed the study’s heterogeneity. Since the heterogeneity was small, the fixed-effects model was used (p > 0.1 or I2 ≤ 50%). Funnel plots were used to evaluate publication bias.

### Certainty of evidence assessment

GRADEpro Guideline Development Tool software (Evidence Prime, Inc, Seattle, WA) was used to create a summary of the findings table to assess the strength of the evidence. Individual GRADE criteria were considered, and evidence certainty was calculated. The GRADE system evaluates evidence certainty as high, moderate, low, or very low [31].

## Results

### Study selection

**Fig 1:**
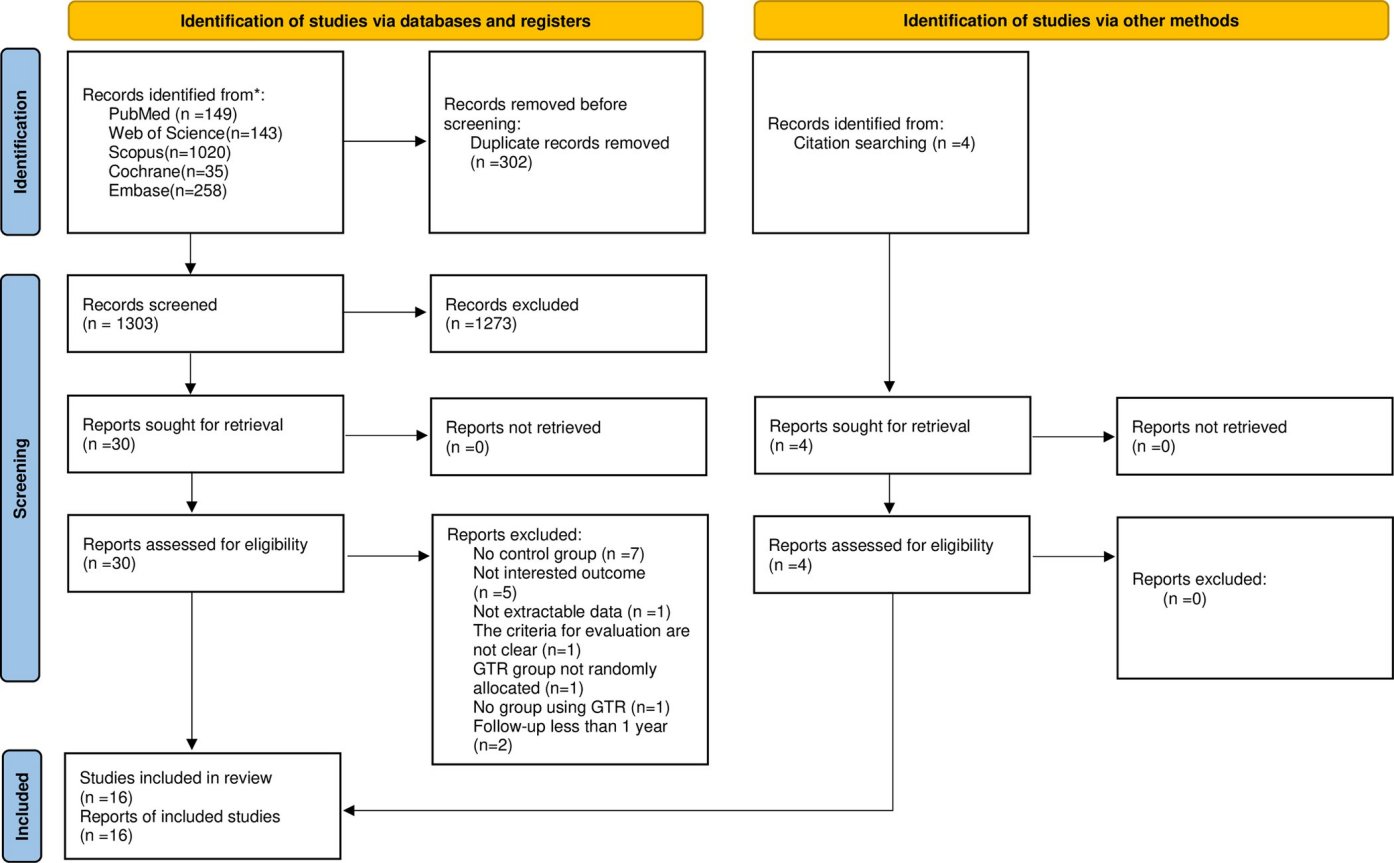
PRISMA statement 2020 flow diagram.

EndNote X21 received 1605 records from different electronic databases. After removing the duplicate publications, a total of 1273 were excluded based on their title and abstract, resulting in 30 papers being considered for full-text review. All studies identified after excluding duplications are shown in S2 Table. In parallel, 4 references were identified through manual searches. Next, 34 papers were full-text reviewed for eligibility, 18 studies [32–49] were excluded for different reasons (S3 Table). Finally, 16 studies [50–65] met the inclusion criteria and were included in the meta-analysis (Fig 1). Three investigators, N.M., X.G., and B.S., conducted all the search processes and examined all the studies separately. In cases of disagreement, a fourth reviewer, A.L., was consulted in order to reach an agreement.

### Study characteristics

All data extracted in primary studies is shown in S4 Table. Table 1 summarizes the main characteristics of each of the included studies. All papers included in the analysis were RCTs published after 2000, except for Pecora et al. [59]. The intervention groups showed 381 lesions, whereas the control groups had 309 lesions. All studies included a control group that did not use GTR procedures and an experimental group that utilized GTR. All studies analyzed 2D radiographic healing based on the criteria defined by Molven et al. or Rud et al. [6, 7], and four studies [50, 51, 53, 57] investigated 3D radiographic healing using the modified PENN 3D criteria.

**Table 1:** Characteristics of included studies.

Author/year	Lesion type	GTR		Intervention group (Ex. G)			Control group (Cont. G)		
		Technique	Materials	Cases	success	Failure	Cases	Success	Failure
Albagle et al. 2023 [50]	Confined	BG	Collagen-based BG	32	29	3	26	24	2
Arpitha M et al. 2023 [51]	Through and through	BG + APCs	Collagen-based BG mixed with i-PRF	18	18	0	16	14	2
Chen &Shen 2016 [52]	Confined	BG + Mb	Bovine bone Hap + Collagen Mb.	17	17	0	19	18	1
	Through and through			25	23	2	19	13	6
Dhamija et al. 2020 [53]	Through and through	APCs	PRP	16	15	1	16	15	1
Dhiman et al. 2015 [54]	Apico-marginal confined to the buccal aspect of the root	APCs	PRF as Mb	15	13	2	15	12	3
Dominiak et al. 2009 [55]	Mean = width 8.38mm, height 9mm, depth 8.40 mm3(Confined)	Mb	Collagen Mb.	26	21	5	25	16	9
		BG	Bovine-derived Hap	30	25	5			
		BG + APCs as Mb	Bovine-derived Hap + PRP	25	23	2			
Pan et al. 2011 [56]	EX. G = (14.77 +- 4.62 mm), Cont. G = (13.31+-5.51 mm)	BG + Mb	Bovine bone Hap + Collagen Mb.	22	21	1	21	19	2
Parmar et al. 2019 [57]	D≥10mm &through and through	Mb	Collagen Mb.	15	15	0	15	14	1
Pecora et al. 1995 [59]	D≥10mm &through and through	Non-resorbable Mb	e-PTFE Mb	10	10	0	10	10	0
Pecora et al. 2001 [58]	D≥10mm &through and through	BG	Calcium sulfate	10	9	1	10	8	2
Rohilla et al. 2022 [60]	Apico-marginal confined to the buccal aspect of the root	Mb	Collagen Mb.	12	10	2	8	8	0
Taschieri et al. 2007 [61]	4 wall Defect(confined)	BG + Mb	Bovine bone Hap + Collagen Mb.	16	14	2	22	18	4
	D≥10mm &through and through			8	6	2	13	8	5
Taschieri et al. 2008a [62]	D≥10mm &through and through	BG + Mb	Bovine bone Hap + Collagen Mb.	17	15	2	14	8	6
Taschieri et al. 2008b [63]	4 wall Defect (confined)	BG + Mb	Bovine bone Hap + Collagen Mb.	16	14	2	22	18	4
	D≥10mm &through and through			17	15	2	14	9	5
Tobon et al. 2002 [64]	0.04–506 mm2 (confined)	Non-resorbable Mb	e-PTFE Mb	9	7	2	9	8	1
		BG + Mb	Synthetic bioactive graft Hap + e-PTFE	8	8	0			
Wang et al. 2017 [65]	25–640 mm2	BG + Mb	Bovine bone Hap + Collagen Mb.	17	15	2	15	11	4

GTR, Guided tissue regeneration; Ex. G, Experimental group; Cont. G, Control group; BG: Bone Graft; Mb, Membrane; APCs, Autologous Platelet Concentrates; Hap, Hydroxyapatite; e-PTFE, expanded polytetrafluoroethylene; i-PRF, injectable platelet-rich fibrin; PRF, platelet-rich fibrin; PRP, platelet-rich plasma

### Quality assessment

**Fig 2:**
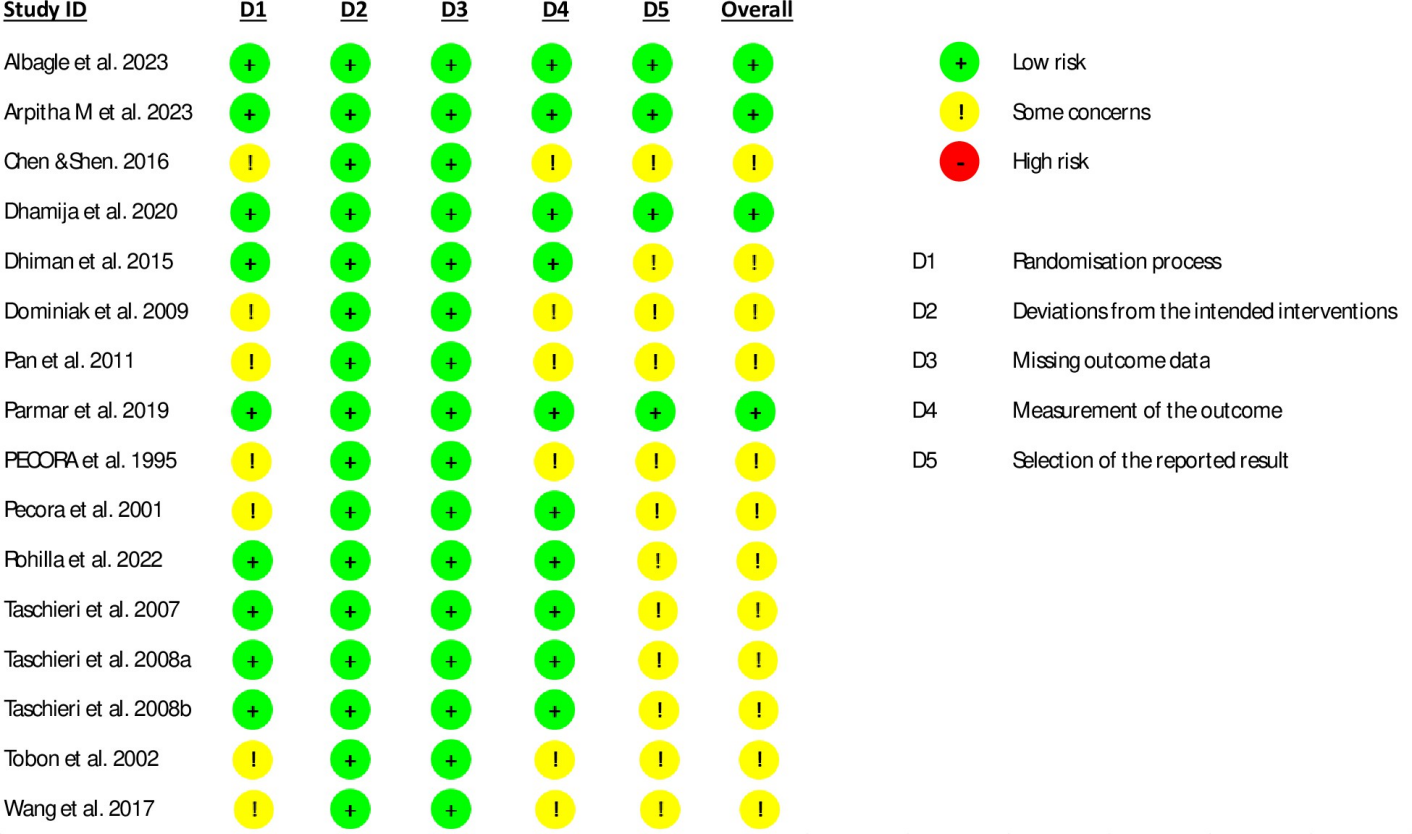
Risk of bias assessment.

The risk of bias in all trials is presented in (Fig 2). However, the randomization process is not well explained in seven studies [52, 55, 56, 58, 59, 64, 65]. Deviations from intended interventions and missing outcome data were assessed as low risk for all studies. Outcome measurement was considered to have some concerns in six studies [52, 55, 56, 59, 64, 65]. Bias due to selective reporting was considered some concern in twelve studies [52, 54–56, 58–65]. Overall, four studies [50, 51, 53, 57] show a low risk of bias, and the remaining twelve studies [52, 54–56, 58–65] exhibit some concerns regarding risk of bias. Completed risk of bias assessments are shown in S5 Table.

### Meta-analysis

**Fig 3:**
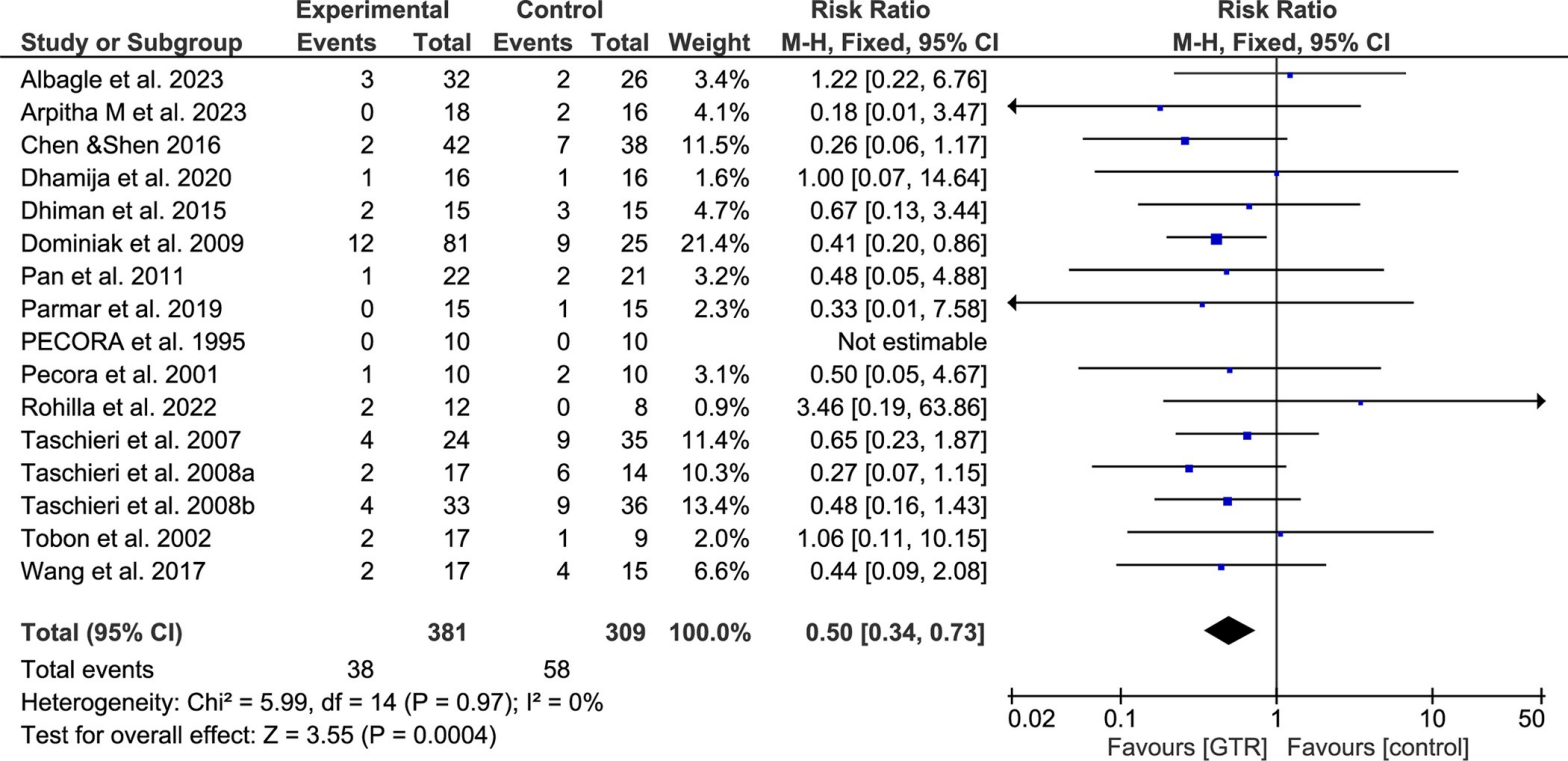
Forest plot and meta-analysis results for 2D evaluation. "Events" indicate failure cases.

**Fig 4:**
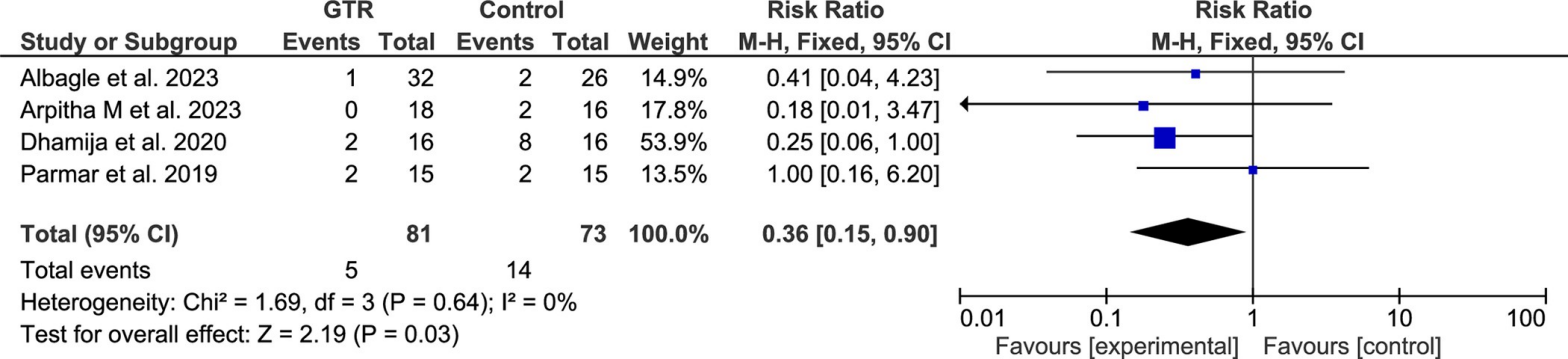
Forest plot and meta-analysis results for 3D evaluation. "Events" indicate failure cases.

Due to the lack of noticeable heterogeneity within the studies included, the meta-analysis used a fixed-effects model. The meta-analysis for all included studies reported the failure rate according to 2D evaluation. The results showed that using the GTR following SRCT significantly improved the healing process compared to the conventional SRCT (RR: 0.50; 95% CI, 0.34–0.73; P < 0.001) (Fig 3). Four studies [50, 51, 53, 57] reported the failure rate according to 3D evaluation. The results showed that using GTR following SRCT significantly improved the healing process compared to the conventional SRCT (RR: 0.36; 95% CI, 0.15–0.90; P < 0.001) (Fig 4).

### Subgroup analysis based on the techniques and materials

**Fig 5:**
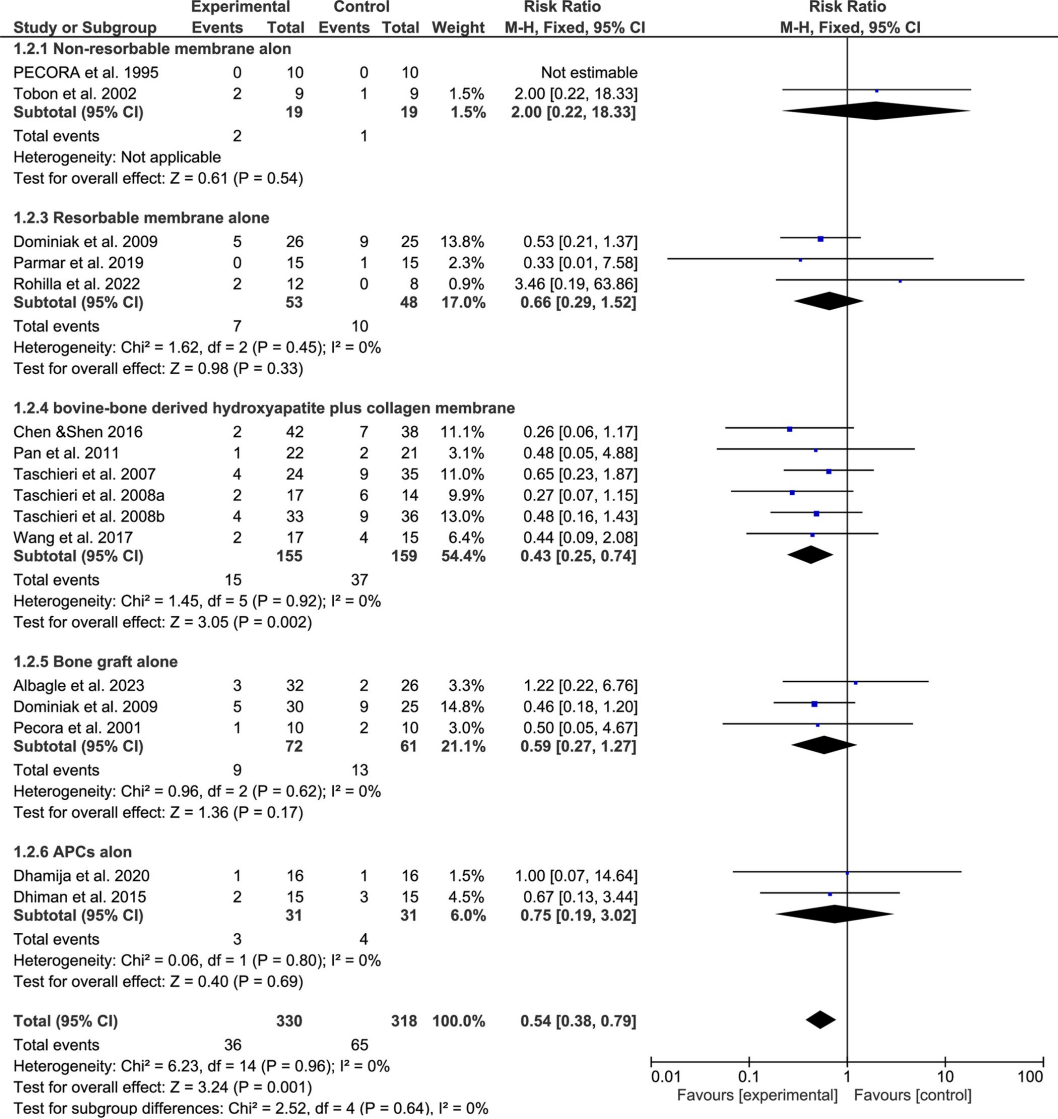
Forest plot and Subgroup analysis based on the GTR technique and materials. "Events" indicate failure cases.

When e-PTFE membranes were used alone, no significant effects were seen in the healing process following SRCT (RR: 2.00; 95% CI, 0.22–18.33; P = 0.54). When resorbable collagen membranes were used alone, the results showed better outcomes but were statistically not significantly different from the control group (RR: 0.66, 95% CI: 0.29–1.52, p = 0.33). In groups that received only bone grafts or APCs, outcomes were also slightly better but not significantly different (bone grafts: RR: 0.59; 95% CI, 0.27–1.27; P = 0.17; APCs: RR: 0.75; 95%, 0.19–3.02; P = 0.69). When bovine bone-derived hydroxyapatite with collagen membrane was used in combination, the success rate was significantly increased compared to the control group (RR: 0.43, 95% Cl: 0.25–0.74, p < 0.001) (Fig 5).

### Subgroup analysis based on the lesion type

**Fig 6:**
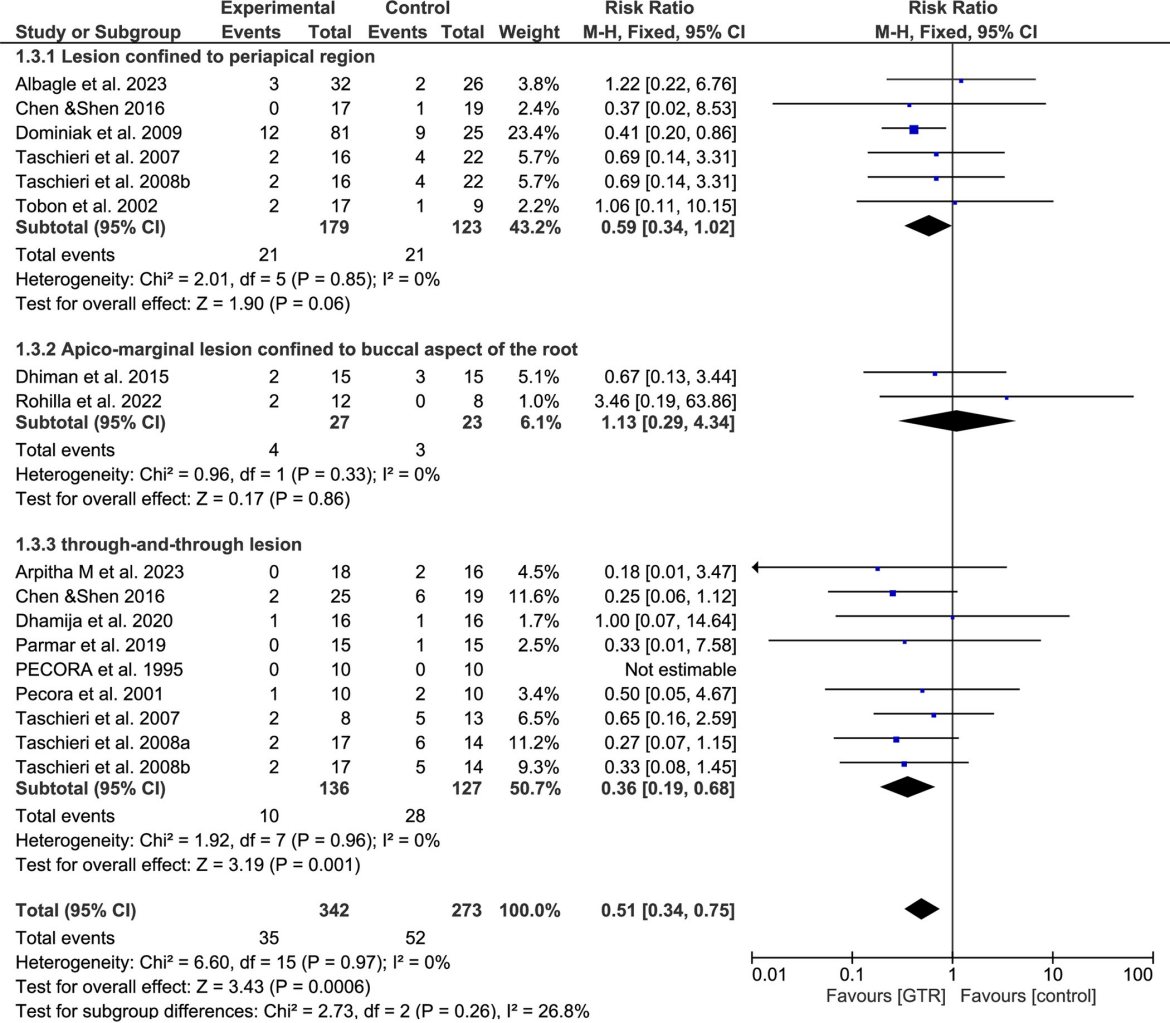
Forest plot and subgroup analysis based on lesion type. "Events" indicate failure cases.

Six studies utilized GTR procedures on confined periapical lesions; there was an advantage towards improved outcomes in GTR groups, without statistically significant difference (RR: 0.59; 95% CI, 0.34–1.02; P = 0.06). When GTR is used on the apico-marginal defect with complete root exposure on the buccal side, there is no significant impact on the healing process after SRCT (RR: 2.00; 95% CI, 0.22–18.33; P = 0.54). When the GTR procedures were used on patients with through-and-through lesions, the results indicated a significant increase in the success rate of the GTR group compared to the control group (RR: 0.36, 95% Cl: 0.19–0.68, p < 0.001) (Fig 6).

### Publication bias

**Fig 7:**
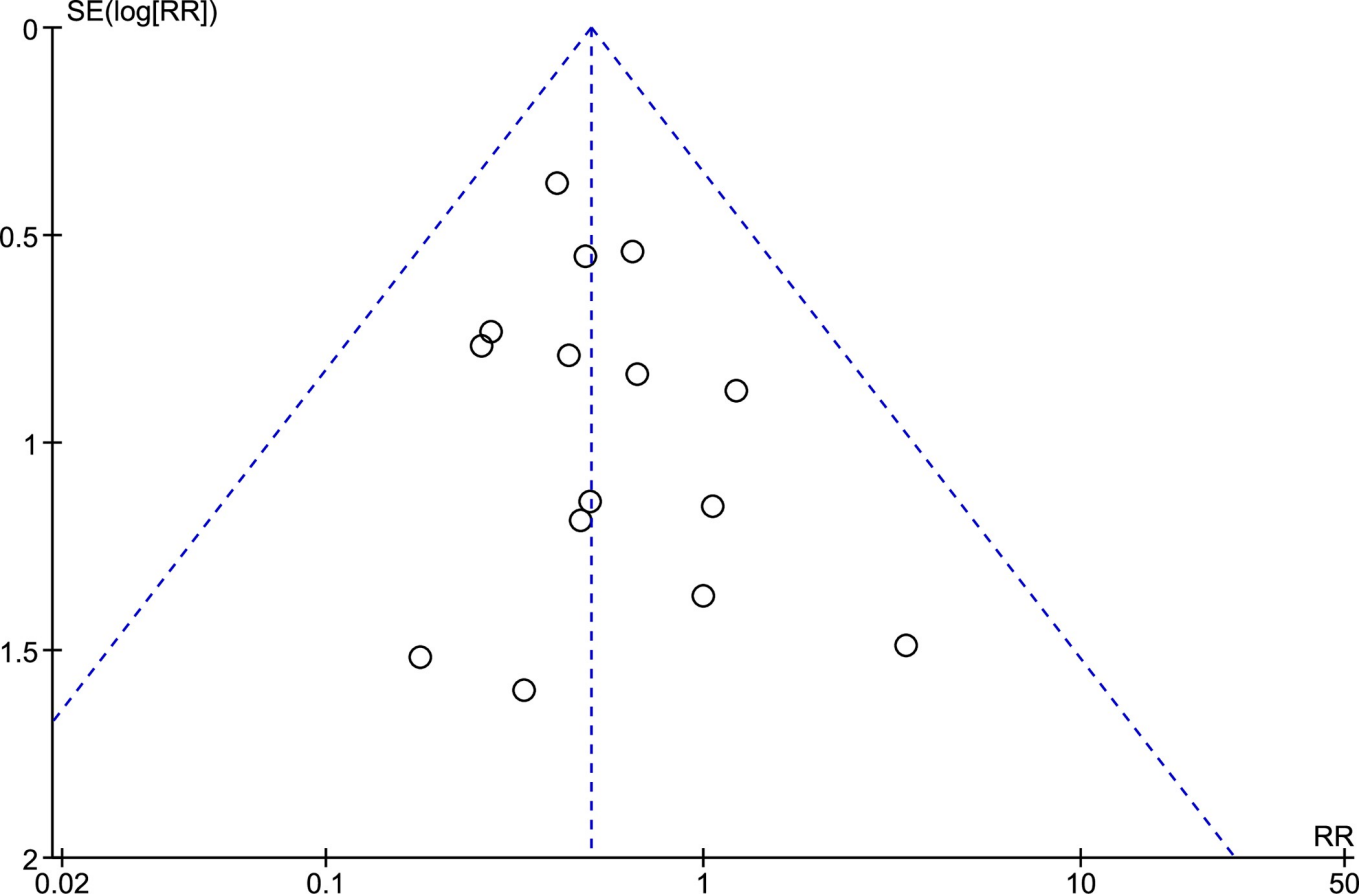
The funnel plot of publication bias.

A funnel plot was created to evaluate the presence of publication bias. The findings indicated that the funnel plot showed bilateral symmetry, indicating the absence of notable publication bias (Fig 7).

### Certainty of evidence

We applied the GRADE process to rank the confidence level of the evidence obtained through our meta-analysis that evaluated the effect of GTR procedures on SRCT (Table 2). After considering the serious risk of bias domain and the non-serious indirectness, imprecision, and inconsistency domains, the success rate of GTR procedures following SRCT is considered to have a moderate grade of evidence.

**Table 2:** Certainty of evidence according to GRADE.

**[GTR] compared to [non-GTR] for [SRCT]**						
**Patient or population:** [patients having SRCT]						
**Intervention:** [GTR]						
**Comparison:** [non-GTR]						
Outcomes	Anticipated absolute effects^*^ (95% CI)		Relative effect (95% CI)	№ of participants (studies)	Certainty of the evidence (GRADE)	Comments
	**Risk with [non-GTR]**	**Risk with [GTR]**				
Healing process after SRCT	19 per 100	**9 per 1**00	**RR 0.50**	690	⨁⨁⨁◯	The GTR procedure likely reduces the failure rate following SRCT.
		(6 to 7)	(0.34 to 0.73)	(16 RCTs)	Moderate^a,b,c,d^	
*The risk in the intervention group (and its 95% confidence interval) is based on the assumed risk in the comparison group and the **relative effect** of the intervention (and its 95% CI).						
**CI:** Confidence Interval; **RR:** Risk Ratio						
**GRADE Working Group grades of evidence**						
**High certainty:** We are very confident that the true effect lies close to that of the estimate of the effect.						
**Moderate certainty:** we are moderately confident in the effect estimate; the true effect is likely to be close to the estimate of the effect, but there is a possibility that it is substantially different.						
**Low certainty:** our confidence in the effect estimate is limited: the true effect may be substantially different from the estimate of the effect.						
**Very low certainty:** we have very little confidence in the effect estimate: the true effect is likely to be substantially different from the estimate of effect.						

a, ’Som concern" risk of bias with allocation concealment, measurement of outcome, and selective reporting; B, Visual inconsistency and statistical analysis show no heterogeneity; c, Direct comparison; d, Narrow confidence interval.

## Discussion

GTR procedures have been used to enhance bone regeneration in SRCT [66]. However, different techniques and biomaterials lead to conflicting outcomes [67], which are still controversial. The current systematic review and meta-analysis include RCTs, which aim to investigate the effects of GTR procedures on the outcome of SRCT. Because there is no outcome difference between the one-year and four-year follow-ups, the studies with more than a one-year follow-up were reported, and the data was explicitly extracted based on the one-year follow-up [68]. Still, it is essential to be careful when generalizing the study’s findings regarding the long-term surgical outcomes of SRCT. The current meta-analysis shows that GTR procedures significantly improve the healing process one year after SRCT, regardless of the evaluation method used. However, results varied in subgroup analysis. Using e-PTFE membranes alone did not improve outcomes, but using collagen membranes, bone grafts, or APCs alone may accelerate healing. Using collagen membranes and bovine bone-derived hydroxyapatite together showed significant improvements. Furthermore, results varied according to the lesion type. GTR treatment for apico-marginal lesions did not improve outcomes, but may accelerate the healing in confined lesions and significantly improve the healing in through-and-through lesions. This meta-analysis was conducted on various types of permanent teeth with different lesion sizes, focusing on lesions caused by endodontic problems. Therefore, the results may apply to patients of different ages with permanent teeth. However, they may not apply to patients with combined Endo-Perio lesions.

The first barrier membrane employed in SRCT as a GTR technique was e-PTFE [69]. This membrane prevents soft tissue growth inside defective areas following SRCT and promotes osteoblast development [70]. However, only two studies [59, 64] in our analysis used this approach, and no evidence justifies its use in SRCT. In contrast, Yoshikawa et al. [71] conducted a study on beagle dogs. They found that using the e-PTFE membrane significantly increased the formation of new cortical bone compared to the control group. Furthermore, using e-PTFE may lead to complications such as membrane exposure and bacterial infection [72] because it is non-absorbable and requires surgical extraction, which increases patient suffering, treatment expenses, and possible issues [73]. The e-PTFE membrane was recently withdrawn from use in SRCT. However, there are alternate materials available [74].

Collagen membranes are the later generation of absorbable membranes and offer several advantages over non-absorbable membranes, including cost-effectiveness and reduced risk of complications [69, 73]. In the present study, absorbable collagen membranes were included in 3 RCTs [55, 57, 60], and no significant differences were observed compared to the control group. Our findings support using collagen membranes, with no statistically significant differences observed compared to the control group. Our finding is consistent with Dominika et al. [55], who also observed a higher success rate after 6 months of follow-up when using collagen membranes. However, after 12 months, there was no significant difference in success rates. Their study showed that using collagen membranes can accelerate the healing process after SRCT. The collagen membrane has varying resorption times, and it is essential for the optimal resorption time to match the time for bone regeneration. Cross-linked technology has been utilized to extend the resorption time and facilitate successful healing [75]. Hence, the collagen membrane should remain in place for a long time.

Using Collagen membranes alone can be unstable, which causes collapse under loads and delayed bone tissue regeneration [76]. To address this issue, some authors have used bone grafts [77]. Bovine bone-derived hydroxyapatite is commonly used because it is a biocompatible graft material with osteoconductive characteristics [78]. The slow resorption rate of bovine bone-derived hydroxyapatite is a critical advantage, as it enables better integration and acts as an effective osteoconductive grafting material during the natural healing process. This can ultimately lead to successful bone healing outcomes [79]. In this study, 6 RCTs [52, 55, 61–63, 65] utilized collagen membranes and bovine bone-derived hydroxyapatite grafts, and we found that this combination significantly improved healing following SRCT. Similar results were reported by Wang et al.[65], who found a significantly better success rate after 12 months of follow-up using collagen membranes combined with bovine bone-derived hydroxyapatite graft.

The bone graft is the most common GTR procedure utilized [12]. In this study, three studies [50, 55, 58] used three different types of bone grafts without additional materials, and we found that this may result in improved healing following SRCT, but without a statistically significant difference. However, Sreedevi et al. [80] found that using freeze-dried hydroxyapatite bone graft material resulted in successful bone healing compared to the control group. The different types of bone grafts have varying success levels and potential advantages and disadvantages [12]. Autogenous bone grafts are the only type that has osteogenesis, osteoinduction, and osteoconduction properties, with a success rate of 95% [15]; however, due to their drawbacks, such as postoperative pain, longer surgery time, and increased morbidity, other graft types are often preferred, such as allograft, xenograft, and alloplastic grafts [81]. Nonetheless, some studies have shown that combining bone grafts with barrier membranes can improve clinical outcomes [82].

Some studies have investigated using APCs to improve healing and regeneration after SRCT [12]. The APCs provide platelets, leukocytes, and growth factors that promote tissue growth and blood flow [83]. Our current meta-analysis found that using APCs resulted in more favorable healing outcomes without a statistically significant difference. Dhamija et al. [53] found a significantly better success rate with PRP in a 3D assessment but no significant difference in a 2D evaluation. The i-PRF is the third generation of APCs [25]. In this analysis, only one study [51] used i-PRF with a collagen-based bone graft. Further research is needed to establish the efficacy of this treatment protocol.

SRCT may involve dealing with many compromised conditions. Based on several clinical and experimental studies, periapical lesions have been categorized into three main types: (1) confined to periapical areas without erosion of the lingual cortex, (2) through-and-through lesions (tunnel), and (3) apico-marginal lesions [4].

In this study, 6 clinical trials [50, 52, 55, 61, 63, 64] assessed several GTR procedures to promote the repair of a confined lesion in the periapical area. Our findings indicate that utilizing GTR procedures can accelerate wound healing, although there is no significant difference in situations of confined periapical lesions. Our finding is consistent with Chen & Shen [52]. They found that the GTR group demonstrated a better success rate than the control group after 6 months, but the difference was not statistically significant after a year.

Apico-marginal lesions can cause epithelial down growth across the denuded root surface after SRCT, which increases the possibility of a recurrent connection between the apical and marginal tissues. Only two studies [54, 60] in our analysis used GTR procedures for apico-marginal lesions with complete exposure on the buccal surface of the root. Our findings indicate that regenerative techniques may not significantly impact outcomes for these lesions. However, a 2023 case series by Baruwa et al. [84] found that endodontic microsurgery combined with GTR can be a highly effective treatment approach for treating apico-marginal lesions. It is important to note that proper diagnosis and procedures are crucial for achieving successful results. Furthermore, as the interest in using APCs for apico-marginal defects grows, better evidence is needed to support their effectiveness. Further well-conducted trials are necessary to fully understand the potential impact of APCs in these cases.

Fast soft tissue proliferation from the facial and lingual sides can hinder bone growth and lead to incomplete healing or scar tissue formation in through-and-through lesions [4]. Furthermore, these through-and-through lesions may offer a pathway for bacterial infection. Therefore, the inclusion of GTR procedures not only have a regenerative function but also play a crucial role in blocking this pathway [51]. This meta-analysis included 9 studies [51–53, 57–59, 61–63] that evaluated the effects of GTR procedures for these lesions and found that they significantly improve wound healing following SRCT. Similar results were reported by Taschieri et al. [63] when using collagen membrane and hydroxyapatite from bovine bone.

### Strength and limitation

This systematic review and meta-analysis only contained RCTs, which provide robust and reliable evidence. Comprehensive inclusion and exclusion criteria were applied to ensure a focused research question and avoid bias in article selection [85]. The search included multiple databases without language or geographical restrictions, increasing the potential for generalization [86]. The study also considered the impact of lesion type on the effectiveness of different GTR procedures. Subgroup analysis can provide clinicians with further insights into choosing the suitable GTR procedure for different lesion types.

There are various limitations to the current study. Most studies raised "some concerns" based on the Cochrane Collaboration tool. In addition, only a single research study incorporated the third generation of APCs. Therefore, meticulously planned clinical trials of superior quality are necessary. Also, only four studies were included that used modified PENN 3D criteria to evaluate the healing process after SRCT. In recent years, CBCT has been used in more clinical studies by using different criteria. More research needs to be carried out to develop straightforward, standardized ways to evaluate the results of 3D radiographs.

## Conclusion

This meta-analysis showed that GTR processes significantly improve healing after SRCT, regardless of evaluation methods, especially when collagen membranes and bovine bone-derived hydroxyapatite are used together. Furthermore, for through-and-through lesions, the GTR procedures significantly improved the healing after SRCT.

## Supporting information

S1 Checklist PRISMA 2020 checklist. (DOCX)

S1 Table Search strategy. (DOCX)

S2 Table Studies identified after excluding duplications. (XLSX)

S3 Table The excluded studies and reasons for exclusion. (DOCX)

S4 Table All data extracted in primary studies. (XLSX)

S5 Table Completed risk of bias assessments. (XLSX)

## References

[1] Siqueira JF, Jr., Rôças IN. The microbiota of acute apical abscesses. J Dent Res. 2009;88(1):61–5. doi: 10.1177/0022034508328124 . 19131319

[2] Gutmann JL, Harrison JW. Posterior endodontic surgery: anatomical considerations and clinical techniques. Int Endod J. 1985;18(1):8–34. doi: 10.1111/j.1365-2591.1985.tb00415.x . 3858237

[3] Tsesis I, Faivishevsky V, Kfir A, Rosen E. Outcome of surgical endodontic treatment performed by a modern technique: a meta-analysis of literature. J Endod. 2009;35(11):1505–11. Epub 20090920. doi: 10.1016/j.joen.2009.07.025 . 19840638

[4] von Arx T, Alsaeed M. The use of regenerative techniques in apical surgery: A literature review. Saudi Dent J. 2011;23(3):113–27. Epub 20110221. doi: 10.1016/j.sdentj.2011.02.004 ; PubMed Central PMCID: PMC3770231. 24151420 PMC3770231

[5] Berman LH, Hargreaves KM. Cohen’s Pathways of the Pulp-E-Book: Cohen’s Pathways of the Pulp-E-Book: Elsevier Health Sciences; 2020.

[6] Rud J, Andreasen JO, Jensen JE. Radiographic criteria for the assessment of healing after endodontic surgery. Int J Oral Surg. 1972;1(4):195–214. doi: 10.1016/s0300-9785(72)80013-9 . 4199168

[7] Molven O, Halse A, Grung B. Observer strategy and the radiographic classification of healing after endodontic surgery. Int J Oral Maxillofac Surg. 1987;16(4):432–9. doi: 10.1016/s0901-5027(87)80080-2 . 3117915

[8] Schloss T, Sonntag D, Kohli MR, Setzer FC. A Comparison of 2- and 3-dimensional Healing Assessment after Endodontic Surgery Using Cone-beam Computed Tomographic Volumes or Periapical Radiographs. J Endod. 2017;43(7):1072–9. Epub 20170517. doi: 10.1016/j.joen.2017.02.007 . 28527841

[9] Lin L, Chen MY, Ricucci D, Rosenberg PA. Guided tissue regeneration in periapical surgery. J Endod. 2010;36(4):618–25. doi: 10.1016/j.joen.2009.12.012 . 20307733

[10] Tsesis I, Rosen E, Taschieri S, Telishevsky Strauss Y, Ceresoli V, Del Fabbro M. Outcomes of surgical endodontic treatment performed by a modern technique: an updated meta-analysis of the literature. J Endod. 2013;39(3):332–9. Epub 20130123. doi: 10.1016/j.joen.2012.11.044 . 23402503

[11] Corbella S, Taschieri S, Elkabbany A, Del Fabbro M, von Arx T. Guided Tissue Regeneration Using a Barrier Membrane in Endodontic Surgery. Swiss Dent J. 2016;126(1):13–25. doi: 10.61872/sdj-2016-01-140 . 26797778

[12] Bashutski JD, Wang HL. Periodontal and endodontic regeneration. J Endod. 2009;35(3):321–8. doi: 10.1016/j.joen.2008.11.023 . 19249588

[13] Rankow HJ, Krasner PR. Endodontic applications of guided tissue regeneration in endodontic surgery. J Endod. 1996;22(1):34–43. doi: 10.1016/S0099-2399(96)80234-2 . 8618084

[14] Wessing B, Lettner S, Zechner W. Guided Bone Regeneration with Collagen Membranes and Particulate Graft Materials: A Systematic Review and Meta-Analysis. Int J Oral Maxillofac Implants. 2018;33(1):87–100. Epub 20170922. doi: 10.11607/jomi.5461 . 28938035

[15] Sakkas A, Wilde F, Heufelder M, Winter K, Schramm A. Autogenous bone grafts in oral implantology-is it still a "gold standard"? A consecutive review of 279 patients with 456 clinical procedures. Int J Implant Dent. 2017;3(1):23. Epub 20170601. doi: 10.1186/s40729-017-0084-4 ; PubMed Central PMCID: PMC5453915. 28573552 PMC5453915

[16] Ausenda F, Rasperini G, Acunzo R, Gorbunkova A, Pagni G. New Perspectives in the Use of Biomaterials for Periodontal Regeneration. Materials (Basel). 2019;12(13). Epub 20190708. doi: 10.3390/ma12132197 ; PubMed Central PMCID: PMC6651816. 31288437 PMC6651816

[17] Artzi Z, Nemcovsky CE, Tal H. Efficacy of porous bovine bone mineral in various types of osseous deficiencies: clinical observations and literature review. Int J Periodontics Restorative Dent. 2001;21(4):395–405. . 11519708

[18] Otero AIP, Fernandes JCH, Borges T, Nassani L, Castilho RM, Fernandes GVO. Sinus Lift Associated with Leucocyte-Platelet-Rich Fibrin (Second Generation) for Bone Gain: A Systematic Review. J Clin Med. 2022;11(7). Epub 20220328. doi: 10.3390/jcm11071888 ; PubMed Central PMCID: PMC8999497. 35407494 PMC8999497

[19] Giannoudis PV, Dinopoulos H, Tsiridis E. Bone substitutes: an update. Injury. 2005;36 Suppl 3:S20–7. doi: 10.1016/j.injury.2005.07.029 . 16188545

[20] Buzalaf MAR, Levy FM. Autologous platelet concentrates for facial rejuvenation. J Appl Oral Sci. 2022;30:e20220020. Epub 20220905. doi: 10.1590/1678-7757-2022-0020 ; PubMed Central PMCID: PMC9444188. 36074433 PMC9444188

[21] Marx RE, Carlson ER, Eichstaedt RM, Schimmele SR, Strauss JE, Georgeff KR. Platelet-rich plasma: Growth factor enhancement for bone grafts. Oral Surg Oral Med Oral Pathol Oral Radiol Endod. 1998;85(6):638–46. doi: 10.1016/s1079-2104(98)90029-4 . 9638695

[22] Dhurat R, Sukesh M. Principles and Methods of Preparation of Platelet-Rich Plasma: A Review and Author’s Perspective. J Cutan Aesthet Surg. 2014;7(4):189–97. doi: 10.4103/0974-2077.150734 ; PubMed Central PMCID: PMC4338460. 25722595 PMC4338460

[23] Miron RJ, Zucchelli G, Pikos MA, Salama M, Lee S, Guillemette V, et al. Use of platelet-rich fibrin in regenerative dentistry: a systematic review. Clin Oral Investig. 2017;21(6):1913–27. Epub 20170527. doi: 10.1007/s00784-017-2133-z . 28551729

[24] Choukroun J, Adda F, Schoeffler C, Vervelle A. Une opportunité en paro-implantologie: le PRF. Implantodontie. 2001;42(55):e62.

[25] Wang X, Zhang Y, Choukroun J, Ghanaati S, Miron RJ. Effects of an injectable platelet-rich fibrin on osteoblast behavior and bone tissue formation in comparison to platelet-rich plasma. Platelets. 2018;29(1):48–55. Epub 20170329. doi: 10.1080/09537104.2017.1293807 . 28351189

[26] Apaydin ES, Torabinejad M. The effect of calcium sulfate on hard-tissue healing after periradicular surgery. J Endod. 2004;30(1):17–20. doi: 10.1097/00004770-200401000-00003 . 14760901

[27] Maguire H, Torabinejad M, McKendry D, McMillan P, Simon JH. Effects of resorbable membrane placement and human osteogenic protein-1 on hard tissue healing after periradicular surgery in cats. J Endod. 1998;24(11):720–5. doi: 10.1016/S0099-2399(98)80161-1 . 9855821

[28] Tsesis I, Rosen E, Tamse A, Taschieri S, Del Fabbro M. Effect of guided tissue regeneration on the outcome of surgical endodontic treatment: a systematic review and meta-analysis. J Endod. 2011;37(8):1039–45. doi: 10.1016/j.joen.2011.05.016 . 21763891

[29] Page MJ, McKenzie JE, Bossuyt PM, Boutron I, Hoffmann TC, Mulrow CD, et al. The PRISMA 2020 statement: an updated guideline for reporting systematic reviews. Bmj. 2021;372:n71. Epub 20210329. doi: 10.1136/bmj.n71 ; PubMed Central PMCID: PMC8005924. 33782057 PMC8005924

[30] Chandler J, Cumpston M, Li T. Cochrane Handbook for Systematic Reviews of Interventions.

[31] Schünemann H, Brożek J, Guyatt G, Oxman A. Handbook for grading the quality of evidence and the strength of recommendations using the GRADE approach. Updated October. 2013;2013:15.

[32] Del Fabbro M, Ceresoli V, Lolato A, Taschieri S. Effect of platelet concentrate on quality of life after periradicular surgery: A randomized clinical study. Journal of Endodontics. 2012;38(6):733–9. doi: 10.1016/j.joen.2012.02.022 PubMed Central PMCID: PMC22595104. 22595104

[33] Dietrich T, Zunker P, Dietrich D, Bernimoulin JP. Periapical and periodontal healing after osseous grafting and guided tissue regeneration treatment of apicomarginal defects in periradicular surgery: results after 12 months. Oral Surg Oral Med Oral Pathol Oral Radiol Endod. 2003;95(4):474–82. doi: 10.1067/moe.2003.39 . 12686934

[34] Gajiwala AL, Kumar BD, Chokhani P. Evaluation of demineralised, freeze-dried, irradiated bone allografts in the treatment of osseous defects in the oral cavity. Cell Tissue Bank. 2007;8(1):23–30. doi: 10.1007/s10561-006-9014-z . 16838195

[35] Garg M, Srivastava V, Chauhan R, Pramanik S, Khanna R. Application of platelet-rich fibrin and freeze-dried bone allograft following apicoectomy: A comparative assessment of radiographic healing. Indian J Dent Res. 2023;34(1):40–4. doi: 10.4103/ijdr.ijdr_810_22 . 37417055

[36] Garrett K, Kerr M, Hartwell G, O’Sullivan S, Mayer P. The effect of a bioresorbable matrix barrier in endodontic surgery on the rate of periapical healing: an in vivo study. Journal of endodontics. 2002;28(7):503–6. doi: 10.1097/00004770-200207000-00003 12126375

[37] Goyal B, Tewari S, Duhan J, Sehgal PK. Comparative evaluation of platelet-rich plasma and guided tissue regeneration membrane in the healing of apicomarginal defects: A clinical study. Journal of Endodontics. 2011;37(6):773–80. doi: 10.1016/j.joen.2011.03.003 21787487

[38] Johri S, Verma P, Tikku AP, Bains R, Kohli N. Effect of amniotic membrane and platelet-rich fibrin membrane on bone healing post endodontic surgery: An ultrasonographic, randomized controlled study. J Tissue Eng Regen Med. 2022;16(12):1208–22. Epub 20221119. doi: 10.1002/term.3362 . 36401577

[39] Karan NB, Aricioğlu B. Assessment of bone healing after mineral trioxide aggregate and platelet-rich fibrin application in periapical lesions using cone-beam computed tomographic imaging. Clin Oral Investig. 2020;24(2):1065–72. Epub 20190718. doi: 10.1007/s00784-019-03003-x . 31321573

[40] Kim E, Song JS, Jung IY, Lee SJ, Kim S. Prospective clinical study evaluating endodontic microsurgery outcomes for cases with lesions of endodontic origin compared with cases with lesions of combined periodontal-endodontic origin. J Endod. 2008;34(5):546–51. Epub 20080306. doi: 10.1016/j.joen.2008.01.023 . 18436032

[41] Marín-Botero ML, Domínguez-Mejía JS, Arismendi-Echavarría JA, Mesa-Jaramillo AL, Flórez-Moreno GA, Tobón-Arroyave SI. Healing response of apicomarginal defects to two guided tissue regeneration techniques in periradicular surgery: A double-blind, randomized-clinical trial. International Endodontic Journal. 2006;39(5):368–77. doi: 10.1111/j.1365-2591.2006.01081.x PubMed Central PMCID: PMC16640635. 16640635

[42] Meschi N, Fieuws S, Vanhoenacker A, Strijbos O, Van der Veken D, Politis C, et al. Root-end surgery with leucocyte- and platelet-rich fibrin and an occlusive membrane: a randomized controlled clinical trial on patients’ quality of life. Clin Oral Investig. 2018;22(6):2401–11. Epub 20180309. doi: 10.1007/s00784-018-2343-z . 29524025

[43] Meschi N, Vanhoenacker A, Strijbos O, Camargo Dos Santos B, Rubbers E, et al. Multi-modular bone healing assessment in a randomized controlled clinical trial of root-end surgery with the use of leukocyte- and platelet-rich fibrin and an occlusive membrane. Clin Oral Investig. 2020;24(12):4439–53. Epub 20200516. doi: 10.1007/s00784-020-03309-1 . 32418011

[44] Sahib AM, Al-Adili SS. Evaluation of healing process of periapical defect filled by platelet rich fibrin using cone beam computed tomography–comparative clinical study. SCOPUS IJPHRD CITATION SCORE. 2019;10(6):466.

[45] Stassen LFA, Hislop WS, Still DM, Moos KF. Use of anorganic bone in periapical defects following apical surgery—A prospective trial. British Journal of Oral and Maxillofacial Surgery. 1994;32(2):83–5. doi: 10.1016/0266-4356(94)90133-3 8199153

[46] Taschieri S, Corbella S, Tsesis I, Del Fabbro M. Impact of the use of plasma rich in growth factors (PRGF) on the quality of life of patients treated with endodontic surgery when a perforation of sinus membrane occurred. A comparative study. Oral Maxillofac Surg. 2014;18(1):43–52. Epub 20130110. doi: 10.1007/s10006-012-0386-x . 23306948

[47] Tikku AP, Kumar S, Loomba K, Chandra A, Verma P, Aggarwal R. Use of ultrasound, color Doppler imaging and radiography to monitor periapical healing after endodontic surgery. Journal of oral science. 2010;52(3):411–6. doi: 10.2334/josnusd.52.411 20881334

[48] Wong AWY, Tsang CSC, Zhang S, Li KY, Zhang C, Chu CH. Treatment outcomes of single-visit versus multiple-visit non-surgical endodontic therapy: A randomised clinical trial. BMC Oral Health. 2015;15(1). doi: 10.1186/s12903-015-0148-x PubMed Central PMCID: PMC26687126. 26687126 PMC4684923

[49] You JS, Jung GW, Oh JS, Moon SY, Lee WP, Jo HH. Volumetric evaluation of effects of platelet-rich fibrin and concentrated growth factor on early bone healing after endodontic microsurgery: a randomized controlled trial. BMC Oral Health. 2023;23(1):821. Epub 20231029. doi: 10.1186/s12903-023-03530-w ; PubMed Central PMCID: PMC10613388. 37899448 PMC10613388

[50] Albagle A, Kohli MR, Kratchman SI, Lee SM, Karabucak B. Periapical healing following endodontic microsurgery with collagen-based bone-filling material: A randomized controlled clinical trial. Int Endod J. 2023;56(12):1446–58. Epub 20230911. doi: 10.1111/iej.13973 . 37695450

[51] Arpitha M, Tewari S, Sangwan P, Gupta A. Efficacy of mixture of injectable-platelet-rich fibrin and type-1 collagen particles on the closure of through-and-through periapical bone defects: A randomized controlled trial. International Endodontic Journal. 2023;56(10):1197–211. doi: 10.1111/iej.13954 WOS:001031786200001. 37418583

[52] CHEN Y, SHEN J Clinical Observation of GTR Combined with Bone Grafting in Endodontic Microsurgery. Journal of Oral Science Research. 2016;32(12):1269.

[53] Dhamija R, Tewari S, Sangwan P, Duhan J, Mittal S. Impact of Platelet-rich Plasma in the Healing of Through-and- through Periapical Lesions Using 2-dimensional and 3-dimensional Evaluation: A Randomized Controlled Trial. Journal of Endodontics. 2020;46(9):1167–84. doi: 10.1016/j.joen.2020.06.004 WOS:000572345400002. 32553878

[54] Dhiman M, Kumar S, Duhan J, Sangwan P, Tewari S. Effect of Platelet-rich Fibrin on Healing of Apicomarginal Defects: A Randomized Controlled Trial. J Endod. 2015;41(7):985–91. Epub 20150508. doi: 10.1016/j.joen.2015.04.004 . 25963290

[55] Dominiak M, Lysiak-Drwal K, Gedrange T, Zietek M, Gerber H. Efficacy of healing process of bone defects after apicectomy: results after 6 and 12 months. J Physiol Pharmacol. 2009;60 Suppl 8:51–5. . 20400792

[56] Pan X, Wang X, Li Y. Application of guided tissue regeneration in apical microsurgery. Chin J Conserv Dent. 2001;21:41–4.

[57] Parmar PD, Dhamija R, Tewari S, Sangwan P, Gupta A, Duhan J, et al. 2D and 3D radiographic outcome assessment of the effect of guided tissue regeneration using resorbable collagen membrane in the healing of through-and-through periapical lesions—a randomized controlled trial. Int Endod J. 2019;52(7):935–48. Epub 20190306. doi: 10.1111/iej.13098 . 30758848

[58] Pecora G, De Leonardis D, Ibrahim N, Bovi M, Cornelini R. The use of calcium sulphate in the surgical treatment of a ’through and through’ periradicular lesion. International Endodontic Journal. 2001;34(3):189–97. doi: 10.1046/j.1365-2591.2001.00369.x 12193264

[59] Pecora G, Kim S, Celletti R, Davarpanah M. The guided tissue regeneration principle in endodontic surgery: one‐year postoperative results of large periapical lesions. International Endodontic Journal. 1995;28(1):41–6. doi: 10.1111/j.1365-2591.1995.tb00155.x PubMed Central PMCID: PMC7642328. 7642328

[60] Rohilla R, Tewari S, Nayyar AS. Efficacy of Guided Tissue Regeneration (GTR) membranes in the healing of apico-marginal defects: A prospective, controlled clinical trial. International Journal of Orofacial Research. 2017;2(1):11–7.

[61] Taschieri S, Del Fabbro M, Testori T, Weinstein R. Efficacy of xenogeneic bone grafting with guided tissue regeneration in the management of bone defects after surgical endodontics. J Oral Maxillofac Surg. 2007;65(6):1121–7. doi: 10.1016/j.joms.2006.10.022 . 17517295

[62] Taschieri S, Fabbro MD, Testori T, Saita M, Weinstein R. Efficacy of guided tissue regeneration in the management of through-and-through lesions following surgical endodontics: A preliminary study. Int J Periodontics Restorative Dent. 2008;28(3):265–71. PubMed Central PMCID: PMC18605602. 18605602

[63] Taschieri S, Testori T, Azzola F, Del Fabbro M, Valentini P. Guided-tissue regeneration in endodontic surgery. Revue de Stomatologie et de Chirurgie Maxillo-Faciale. 2008;109(4):213–7. doi: 10.1016/j.stomax.2007.10.008 18533208

[64] Tobón SI, Arismendi JA, Marïn ML, Mesa AL, Valencia JA. Comparison between a conventional technique and two bone regeneration techniques in periradicular surgery. International Endodontic Journal. 2002;35(7):635–41. doi: 10.1046/j.1365-2591.2002.00523.x PubMed Central PMCID: PMC12190904. 12190904

[65] Wang K, Gui L, Lu H, He X, Li D, Liu C, et al. Efficacy and safety of pembrolizumab with preoperative neoadjuvant chemotherapy in patients with resectable locally advanced head and neck squamous cell carcinomas. Frontiers in Immunology. 2023;14. doi: 10.3389/fimmu.2023.1189752 37583698 PMC10425220

[66] Mastromihalis N, Goldstein S, Greenberg M, Friedman S. Applications for guided bone regeneration in endodontic surgery. N Y State Dent J. 1999;65(5):30–2. . 10409840

[67] Nyman S, Lindhe J, Karring T, Rylander H. New attachment following surgical treatment of human periodontal disease. J Clin Periodontol. 1982;9(4):290–6. doi: 10.1111/j.1600-051x.1982.tb02095.x . 6964676

[68] Rubinstein RA, Kim S. Long-term follow-up of cases considered healed one year after apical microsurgery. J Endod. 2002;28(5):378–83. doi: 10.1097/00004770-200205000-00008 . 12026924

[69] Zhang M, Zhou Z, Yun J, Liu R, Li J, Chen Y, et al. Effect of Different Membranes on Vertical Bone Regeneration: A Systematic Review and Network Meta-Analysis. Biomed Res Int. 2022;2022:7742687. Epub 20220714. doi: 10.1155/2022/7742687 ; PubMed Central PMCID: PMC9303140. 35872861 PMC9303140

[70] Wang J, Wang L, Zhou Z, Lai H, Xu P, Liao L, et al. Biodegradable Polymer Membranes Applied in Guided Bone/Tissue Regeneration: A Review. Polymers (Basel). 2016;8(4). Epub 20160329. doi: 10.3390/polym8040115 ; PubMed Central PMCID: PMC6431950. 30979206 PMC6431950

[71] Yoshikawa G, Murashima Y, Wadachi R, Sawada N, Suda H. Guided bone regeneration (GBR) using membranes and calcium sulphate after apicectomy: a comparative histomorphometrical study. Int Endod J. 2002;35(3):255–63. doi: 10.1046/j.1365-2591.2002.00473.x . 11985677

[72] Calciolari E, Akcalı A, Donos N. The role of osteopromotive membranes in guided bone regeneration. Bone Augmentation by Anatomical Region: Techniques and Decision‐Making. 2020:69–93.

[73] Hämmerle CH, Jung RE. Bone augmentation by means of barrier membranes. Periodontol 2000. 2003;33:36–53. doi: 10.1046/j.0906-6713.2003.03304.x . 12950840

[74] Rakhmatia YD, Ayukawa Y, Furuhashi A, Koyano K. Current barrier membranes: titanium mesh and other membranes for guided bone regeneration in dental applications. J Prosthodont Res. 2013;57(1):3–14. Epub 20130121. doi: 10.1016/j.jpor.2012.12.001 . 23347794

[75] Park WB, Crasto GJ, Han JY, Kang P, Lim HC. Bone Regenerative Potential of Cross-Linked Collagen Membrane in Peri-Implant Osseous Defect: Case Series with Histologic/Micro-Computed Tomographic Findings. Medicina (Kaunas). 2023;59(1). Epub 20230115. doi: 10.3390/medicina59010176 ; PubMed Central PMCID: PMC9861024. PMC9861024 36676800

[76] Bornert F, Herber V, Sandgren R, Witek L, Coelho PG, Pippenger BE, et al. Comparative barrier membrane degradation over time: Pericardium versus dermal membranes. Clin Exp Dent Res. 2021;7(5):711–8. Epub 20210505. doi: 10.1002/cre2.414 ; PubMed Central PMCID: PMC8543466. 33949796 PMC8543466

[77] Hürzeler MB, Kohal RJ, Naghshbandi J, Mota LF, Conradt J, Hutmacher D, et al. Evaluation of a new bioresorbable barrier to facilitate guided bone regeneration around exposed implant threads. An experimental study in the monkey. Int J Oral Maxillofac Surg. 1998;27(4):315–20. doi: 10.1016/s0901-5027(05)80623-x . 9698183

[78] Castilla Bolaños MA, Buttigieg J, Briceño Triana JC. Development and characterization of a novel porous small intestine submucosa-hydroxyapatite scaffold for bone regeneration. Mater Sci Eng C Mater Biol Appl. 2017;72:519–25. Epub 20161215. doi: 10.1016/j.msec.2016.11.113 . 28024616

[79] Zhao R, Yang R, Cooper PR, Khurshid Z, Shavandi A, Ratnayake J. Bone Grafts and Substitutes in Dentistry: A Review of Current Trends and Developments. Molecules. 2021;26(10). Epub 20210518. doi: 10.3390/molecules26103007 ; PubMed Central PMCID: PMC8158510. 34070157 PMC8158510

[80] Sreedevi P, Varghese N, Varugheese JM. Prognosis of periapical surgery using bonegrafts: A clinical study. J Conserv Dent. 2011;14(1):68–72. doi: 10.4103/0972-0707.80743 ; PubMed Central PMCID: PMC3099119. 21691510 PMC3099119

[81] Fernandes GVO, Castro F, Pereira RM, Teixeira W, Gehrke S, Joly JC, et al. Critical-size defects reconstruction with four different bone grafts associated with e-PTFE membrane: A histomorphometric experimental in vivo study. Clin Oral Implants Res. 2024;35(2):167–78. Epub 20231121. doi: 10.1111/clr.14210 . 37987205

[82] Basler T, Naenni N, Schneider D, Hämmerle CHF, Jung RE, Thoma DS. Randomized controlled clinical study assessing two membranes for guided bone regeneration of peri-implant bone defects: 3-year results. Clin Oral Implants Res. 2018;29(5):499–507. Epub 20180415. doi: 10.1111/clr.13147 . 29656495

[83] Shirbhate U, Bajaj P. Third-Generation Platelet Concentrates in Periodontal Regeneration: Gaining Ground in the Field of Regeneration. Cureus. 2022;14(8):e28072. Epub 20220816. doi: 10.7759/cureus.28072 ; PubMed Central PMCID: PMC9477433. 36127983 PMC9477433

[84] Baruwa Ao Bds P, Martins Jnr Dds MP, Pires Md Dds M, Pereira B Dds M, Cruz Pm Dds M, Ginjeira A Md DMDP. Management of Apico-marginal Defects With Endodontic Microsurgery and Guided Tissue Regeneration: A Report of Thirteen Cases. J Endod. 2023;49(9):1207–15. Epub 20230717. doi: 10.1016/j.joen.2023.07.009 . 37468061

[85] Burns PB, Rohrich RJ, Chung KC. The levels of evidence and their role in evidence-based medicine. Plast Reconstr Surg. 2011;128(1):305–10. doi: 10.1097/PRS.0b013e318219c171 ; PubMed Central PMCID: PMC3124652. 21701348 PMC3124652

[86] Morrison A, Polisena J, Husereau D, Moulton K, Clark M, Fiander M, et al. The effect of English-language restriction on systematic review-based meta-analyses: a systematic review of empirical studies. Int J Technol Assess Health Care. 2012;28(2):138–44. doi: 10.1017/S0266462312000086 . 22559755

