# Supplementary material for: Impact of regenerative procedure on the healing process following surgical root canal treatment: A systematic review and meta-analysis
Source: PLoS One. 2025 Jan 2;20(1):e0312751. doi: 10.1371/journal.pone.0312751 (PMC11695025; doi:10.1371/journal.pone.0312751)
Supplement: S1 Table — (DOCX) [file pone.0312751.s002.docx]

| **Database** | **Search strategy** |
| --- | --- |
| PubMed | ("apicoectomy"[MeSH Terms] OR "apicoectomy"[All Fields] OR "apicectomies"[All Fields] OR "apicectomy"[All Fields] OR "surgical Root Canal Treatment"[All Fields] OR "root end resection"[All Fields] OR "micro endodontic surgery"[All Fields] OR "root end surgery"[All Fields] OR "apical surgery"[All Fields] OR "periapical surgery"[All Fields] OR "peri radicular surgery"[All Fields]) AND ("Regenerative material"[All Fields] OR "regenerative therapy"[All Fields] OR "regenerative treatment"[All Fields] OR "regenerative surgery"[All Fields] OR "Guided tissue regeneration"[All Fields] OR "bone substitute"[All Fields] OR "periodontal membrane"[All Fields] OR "bone grafting"[All Fields] OR "growth factors"[All Fields] OR "Platelet-rich Fibrin"[All Fields] OR "Titanium-prepared"[All Fields] OR "Platelet-rich Fibrin"[All Fields] OR "autologous platelet concentrates"[All Fields] OR "GTR"[All Fields] OR "PRF"[All Fields] OR "T-PRF"[All Fields] OR "enamel matrix"[All Fields]=**149** |
| Cochrane Library | 1 (Apicectomy OR "surgical Root Canal Treatment" OR "root end resection" OR "micro endodontic surgery" OR "root end surgery" OR "apical surgery" OR "periapical surgery" OR "peri radicular surgery”) = 151  #2 ("Regenerative material" OR "regenerative therapy" OR "regenerative treatment" OR "regenerative surgery" OR "Guided tissue regeneration" OR "bone substitute" OR "periodontal membrane" OR "bone grafting" OR "growth factors" OR "Platelet-rich Fibrin" OR "Titanium-prepared" OR "Platelet-rich Fibrin" OR "autologous platelet concentrates" OR GTR OR PRF OR T-PRF OR "enamel matrix”) =7760  #1 AND #2= **35** |
| Web of Science | TS= ((Apicectomy OR "surgical Root Canal Treatment" OR "root end resection" OR "micro endodontic surgery" OR "root end surgery" OR "apical surgery" OR "periapical surgery" OR "peri radicular surgery")) =1590  TS= (("Regenerative material" OR "regenerative therapy" OR "regenerative treatment" OR "regenerative surgery" OR "Guided tissue regeneration" OR "bone substitute" OR "periodontal membrane" OR "bone grafting" OR "growth factors" OR "Platelet-rich Fibrin" OR "Titanium-prepared" OR "Platelet-rich Fibrin" OR "autologous platelet concentrates" OR GTR OR PRF OR T-PRF OR "enamel matrix")) =263,026  #1 AND #2=**143** |
| Scopus | ( ALL ( ( apicectomy OR "surgical Root Canal Treatment" OR "root end resection" OR "micro endodontic surgery" OR "root end surgery" OR "apical surgery" OR "periapical surgery" OR "peri radicular surgery" ) ) AND ALL ( ( "Regenerative material" OR "regenerative therapy" OR "regenerative treatment" OR "regenerative surgery" OR "Guided tissue regeneration" OR "bone substitute" OR "periodontal membrane" OR "bone grafting" OR "growth factors" OR "Platelet-rich Fibrin" OR "Titanium-prepared" OR "Platelet-rich Fibrin" OR "autologous platelet concentrates" OR GTR OR PRF OR t-PRF OR "enamel matrix" ) ) )=**1020** |
| Embase | #1 (apicectomy OR 'surgical root canal treatment' OR 'root end resection'/exp OR 'root end resection' OR 'micro endodontic surgery' OR 'root end surgery'/exp OR 'root end surgery' OR 'apical surgery' OR 'periapical surgery'/exp OR 'periapical surgery' OR 'peri radicular surgery’) =1,352  #2('regenerative material' OR 'regenerative therapy'/exp OR 'regenerative therapy' OR 'regenerative treatment' OR 'regenerative surgery'/exp OR 'regenerative surgery' OR 'guided tissue regeneration'/exp OR 'guided tissue regeneration' OR 'bone substitute'/exp OR 'bone substitute' OR 'periodontal membrane'/exp OR 'periodontal membrane' OR 'bone grafting'/exp OR 'bone grafting' OR 'growth factors'/exp OR 'growth factors' OR 'titanium-prepared' OR 'platelet-rich fibrin'/exp OR 'platelet-rich fibrin' OR 'autologous platelet concentrates' OR gtr OR prf OR 't prf' OR 'enamel matrix')=534,136  #1 AND #2=**258** |

**S1 Table. Search strategy**
