# Supplementary material for: Impact of regenerative procedure on the healing process following surgical root canal treatment: A systematic review and meta-analysis
Source: PLoS One. 2025 Jan 2;20(1):e0312751. doi: 10.1371/journal.pone.0312751 (PMC11695025; doi:10.1371/journal.pone.0312751)
Supplement: S3 Table — (DOCX) [file pone.0312751.s004.docx]

**S2 Table. The Excluded Studies and Reasons for Exclusion**

| **Author, year** | **Title** | **Reason for exclusion** |
| --- | --- | --- |
| Garg et al. 2023 | Application of platelet-rich fibrin and freeze-dried bone allograft following apicoectomy: A comparative assessment of radiographic healing | No control group |
| Johri et al. 2022 | Effect of amniotic membrane and platelet-rich fibrin membrane on bone healing post endodontic surgery: An ultrasonographic, randomized controlled study |  |
| Botero et al. 2006 | Healing response of apicomarginal defects to two guided tissue regeneration techniques in periradicular surgery: A double-blind, randomized-clinical trial |  |
| Goyal et al. 2011 | Comparative evaluation of platelet-rich plasma and guided tissue regeneration membrane in the healing of apicomarginal defects: A clinical study |  |
| Thakur et al. 2023 | Comparative histological evaluation of two PRF formulations (PRF High and PRF Medium) on quality of life and healing outcome of apicomarginal defects: A randomized clinical trial |  |
| Dietrich et al. 2003 | Periapical and periodontal healing after osseous grafting and guided tissue regeneration treatment of apicomarginal defects in periradicular surgery: results after 12 months |  |
| Gajiwala et al., 2007 | Evaluation of demineralised, freeze-dried, irradiated bone allografts in the treatment of osseous defects in the oral cavity |  |
| Nazife Begum Karan & Banu Aricioglu 2020 | Assessment of bone healing after mineral trioxide aggregate and platelet-rich fibrin application in periapical lesions using cone-beam computed tomographic imaging | Non-relevant outcome |
| Garrett et al. 2002 | The effect of a bioresorbable matrix barrier in endodontic surgery on the rate of periapical healing: an in vivo study |  |
| Fabbro et al. 2012 | Effect of platelet concentrate on quality of life after periradicular surgery: A randomized clinical study |  |
| Meschi et al. 2018 | Root-end surgery with leucocyte- and platelet-rich fibrin and an occlusive membrane: a randomized controlled clinical trial on patients' quality of life |  |
| Taschieri et al. 2014 | Impact of the use of plasma rich in growth factors (PRGF) on the quality of life of patients treated with endodontic surgery when a perforation of sinus membrane occurred. A comparative study |  |
| You et al. 2023 | Volumetric evaluation of effects of platelet-rich fibrin and concentrated growth factor on early bone healing after endodontic microsurgery: a randomized controlled trial | Follow-up period less than 1 year |
| Ahmed Mahdi Sahib & Sahar Shakir Al-Adili 2019 | Evaluation of healing process of periapical defect filled by platelet rich fibrin using cone beam computed tomography–comparative clinical study |  |
| Stasscn et al. 1994 | Evaluation of healing process of periapical defect filled by platelet rich fibrin using cone beam computed tomography–comparative clinical study | 9 cases with lateral perforation, the criteria for evaluation not clear |
| Tikku et al. 2010 | Use of ultrasound, color Doppler imaging and radiography to monitor periapical healing after endodontic surgery | No GTR group |
| Meschi et al. 2020 | Multi-modular bone healing assessment in a randomized controlled clinical trial of root-end surgery with the use of leukocyte- and platelet-rich fibrin and an occlusive membrane | The data that could not be extracted |
| Kim et al. 2008 | Prospective clinical study evaluating endodontic microsurgery outcomes for cases with lesions of endodontic origin compared with cases with lesions of combined periodontal-endodontic origin | GTR group that was not assigned randomly |
