# Supplementary material for: Impact of regenerative procedure on the healing process following surgical root canal treatment: A systematic review and meta-analysis
Source: PLoS One. 2025 Jan 2;20(1):e0312751. doi: 10.1371/journal.pone.0312751 (PMC11695025; doi:10.1371/journal.pone.0312751)
Supplement: Attachment — Submitted filename: Response to Reviewers.docx [file pone.0312751.s007.docx]

Response to Reviewers

Prof. Fahad Umer

Academic Editor

PLOS ONE

20 September 2024

Dear Academic Editor [Fahad Umer]

Subject: Submission of revised manuscript No.: PONE-D-24-25494

Impact of Regenerative Procedure on The Healing Process Following Surgical Root Canal Treatment: A systematic review and meta-analysis

We appreciate the opportunity to submit a revised revision of our manuscript. It is greatly appreciated that you and the other reviewers have devoted the time and effort necessary to offer valuable feedback on our manuscript. Also, we thank the reviewers for their insightful feedback regarding our paper. We have successfully implemented modifications to incorporate most of the reviewers' recommendations. We highlighted the modifications in the Revised Manuscript by the red color.

We hope the revised version is appropriate for publishing and look forward to your response.

Additional Editor Comments:

The main concerns are as follows:

Numerous grammatical errors throughout the manuscript

Multiple spelling mistakes

Inconsistent formatting

Response:

Thank you for raising these points. We have already hired a professional proofreader to thoroughly review all sections of the paper as recommended.

Response to Reviewer #1:

Thank you for taking the time to review our paper. Your feedback and suggestions have been valuable in improving the quality of our work.

We have carefully addressed your point and made the necessary modifications as suggested.

1. There are several punctuation errors and some typographical errors all through the manuscript. The use of full stop, comma, semi colon and numbers are used in sentences which changed the context entirely. Advice is to seek a professional English language reviewer.

Response:

Thank you for raising this point. We have already hired a professional proofreader to thoroughly review all sections of the paper as recommended.

2. The formatting of the reference should be checked to ensure it matches the guidelines of the journal.

Response:

Thank you for raising this point. We have re-checked and downloaded the referencing style from the PLoS ONE website to ensure it matches the journal's guidelines.

3. The last sentence of the paragraph states "However, The GTR procedure has no advantage in treating apical marginal lesions." - This statement may not be entirely true as the application of GTR in cases of marginal defects is dependent on the diagnosis and appropriate use of GTR technique. See the case series published in JOE, although a low level of evidence, it provides insight on the use of GTR in apical marginal lesions https://doi.org/10.1016/j.joen.2023.07.009

Response:

Thank you for your valuable suggestions. This sentence was written in the conclusion, so it is crucial to ensure that the conclusion accurately reflects the study's key findings. We have included this point in the discussion part as recommended. (P.15, Line 250).

[However, a 2023 case series by Baruwa et al. found that endodontic microsurgery combined with GTR can be a highly effective treatment approach for treating apico-marginal lesions. It is important to note that proper diagnosis and procedures are crucial for achieving successful results.]

Response to Reviewer #2:

Thank you for taking the time to review our paper. Your feedback and suggestions have been valuable in improving the quality of our work.

We have carefully addressed your points and made the necessary modifications as suggested.

1. Introduction: GTR is comprehensively covered. Add more information about nonsurgical root canals and surgical root canal assessment criteria.

Response:

Thank you for your valuable suggestions. We have included this point in the introduction part as recommended. (p.3, line 27).

[Success in both RCT and SRCT relies on the absence of signs of infection and inflammation, along with radiography showing reduced periapical lesion size and normal growth of the periodontal ligament gap. The evaluation of healing after SRCT is commonly conducted using the criteria established by Rud et al. and Molven et al. on 2D imaging, which categorizes healing as complete, incomplete, uncertain, or unsatisfactory. On the other hand, the Modified PENN criteria have been used to evaluate healing on 3D imaging.]

2. Methods: Data Extraction: Give evidence for the criteria that you used to assess success and failure.

Response:

Thank you for raising this point. The success and failure were established based on the criteria described by Rud et al. and Molven et al. for 2D imaging and modified PENN's criteria described by Schloss et al. for 3D imaging. For statistical purposes, the outcomes were dichotomized into success and failure; this method of dichotomization has been described and used in many Randomized control trials. We clarified that in the Data Extraction section as recommended. (p.6, line 95).

Here are some references that use the same dichotomization method: Schloss et al.[1], Parmer et al.[2], Dhamija et al.[3]

[The clinical outcomes are evaluated by the presence or absence of signs of infection and inflammation. Radiographically, the healing assessment was determined by using the criteria established by Rud et al. or Molven et al. (complete, incomplete, uncertain, or unsatisfactory healing) for 2D imaging evaluation, whereas the modified PENN criteria established by Schloss et al. (complete, limited, uncertain, or unsatisfactory healing) were used for 3D imaging evaluation.

The assessment of success and failure was determined based on a comprehensive evaluation of both clinical and radiological outcomes. For statistical purposes, the outcomes were also dichotomized into success and failure. The Success was assessed by the loss of clinical symptoms and the signs of (complete or incomplete healing) for 2D imaging and (Complete or Limited healing) for 3D imaging. Failure was assessed by the presence of clinical symptoms and/or the occurrence of (uncertain or unsatisfactory healing) for 2D and 3D imaging.]

3. Discussion: 2nd paragraph: What you met by possible issues?

You did not discuss the slow resorption rate of bovine bone-derived hydroxyapatite, or whether it could affect the radiographic findings.

Response:

Thank you for raising this point. This point has been added to the discussion part as recommended. (p.14, line 217).

[The slow resorption rate of bovine bone-derived hydroxyapatite is a critical advantage, as it enables better integration and acts as an effective osteoconductive grafting material during the natural healing process. This can ultimately lead to successful bone healing outcomes.]

References

1.	Schloss T, Sonntag D, Kohli MR, Setzer FC. A Comparison of 2- and 3-dimensional Healing Assessment after Endodontic Surgery Using Cone-beam Computed Tomographic Volumes or Periapical Radiographs. J Endod. 2017;43(7):1072-9. Epub 20170517. doi: 10.1016/j.joen.2017.02.007. PubMed PMID: 28527841.

2.	Parmar PD, Dhamija R, Tewari S, Sangwan P, Gupta A, Duhan J, Mittal S. 2D and 3D radiographic outcome assessment of the effect of guided tissue regeneration using resorbable collagen membrane in the healing of through-and-through periapical lesions - a randomized controlled trial. Int Endod J. 2019;52(7):935-48. Epub 20190306. doi: 10.1111/iej.13098. PubMed PMID: 30758848.

3.	Dhamija R, Tewari S, Sangwan P, Duhan J, Mittal S. Impact of Platelet-rich Plasma in the Healing of Through-and- through Periapical Lesions Using 2-dimensional and 3-dimensional Evaluation: A Randomized Controlled Trial. Journal of Endodontics. 2020;46(9):1167-84. doi: 10.1016/j.joen.2020.06.004. PubMed PMID: WOS:000572345400002.
