# Supplementary material for: Impact of regenerative procedure on the healing process following surgical root canal treatment: A systematic review and meta-analysis
Source: PLoS One. 2025 Jan 2;20(1):e0312751. doi: 10.1371/journal.pone.0312751 (PMC11695025; doi:10.1371/journal.pone.0312751)
Supplement: Attachment — Submitted filename: Response to Reviewers.docx [file pone.0312751.s008.docx]

Response to Reviewers

Prof. Fahad Umer

Academic Editor

PLOS ONE

10 October 2024

Dear Academic Editor [Fahad Umer]

Subject: Submission of revised manuscript No.: PONE-D-24-25494R1

Impact of Regenerative Procedure on The Healing Process Following Surgical Root Canal Treatment: A systematic review and meta-analysis

We appreciate the opportunity to submit a revised revision of our manuscript. It is greatly appreciated that you and the other reviewers have devoted the time and effort necessary to offer valuable feedback on our manuscript. We have successfully implemented modifications to incorporate all of the Editor's recommendations. We utilized the track changes feature in the Word file of the revised manuscript.

We hope the revised version is appropriate for publishing and look forward to your response.

**Editor Comments:**

**Introduction:**

1. It’s a bit prolix and can benefit from making it concise

Response:

Thank you for your suggestion regarding the text's verbosity. We have addressed this issue and made the necessary revisions to ensure the introduction is as concise as recommended. (P.3,4).

**Statistical analysis**

2. Random effect model was not used, so dis-include this.

Response:

Thank you for raising this point. we have noted your suggestion and have removed any references to random effect as recommended. (P.7, Line 223).

**Results:**

**Fig 1: Prisma 2020**

3. Use this flow chart RISMA 2020 flow diagram for new systematic reviews which included searches of databases, registers and other sources (https://www.prisma-statement.org/prisma-2020-flow-diagram) be mindful of formatting and font sizes.

Response:

Thank you for your valuable suggestions. We used the RISMA 2020 flow diagram for new systematic reviews, which included searches of databases, registers, and other sources as recommended.

4. Table 1 is not required since it will be in the Prisma flow diagram and will be redundant information.

Response:

Thank you for your feedback regarding Table 1. We have removed it from the main manuscript as recommended. Instead, we have included it as supporting information. (P.8).

**Discussion**

5. PENN is misspelled as PEEN

Response: Thank you for pointing out the spelling error. We have corrected "PEEN" to "PENN" throughout the manuscript. We appreciate your attention to detail. (P.16, Line 285).

**Conclusion**

6. Modify the conclusion to focus on the statistically significant GTR techniques

Response:

Thank you for your suggestion. We revised and rewrote the conclusion to emphasize the key statistically significant impacts of GTR procedures. (P.16, Line 289).
